# Solvent-Free Synthesis of 2,5-Bis((dimethylamino)methylene)cyclopentanone

**DOI:** 10.3390/mps2030069

**Published:** 2019-08-12

**Authors:** Inês S. Martins, Jaime A. S. Coelho

**Affiliations:** Research Institute for Medicines (iMed.ULisboa), Faculty of Pharmacy, Universidade de Lisboa, 1649-003 Lisbon, Portugal

**Keywords:** dyes, organocatalysis, green chemistry

## Abstract

Available protocols for the synthesis of ketocyanine dyes precursor 2,5-bis((dimethylamino)methylene)cyclopentanone are not straightforward and the reported yields are low to moderate. The important feature in the synthesis of this product through organocatalyzed condensation of cyclopentanone and *N*,*N*-Dimethylformamide dimethyl acetal is the removal of methanol produced during the reaction. By studying the reaction profile, in particular the selectivity for the formation of mono- and bis-condensation products, a high yield of the desired product can be obtained through an operationally simple and solvent-free protocol.

## 1. Introduction

Polymethine dyes have been attracting much attention for high-technology applications, such as photographic sensitization, laser technology, nonlinear optics, optical recording, electronic photography, photovoltaic and solar cells, ion recognition and fluorescence labeling in molecular biology [1,2,3,4]. In particular, ketocyanines are synthetic colored molecules that generally absorb in the visible to near-infrared region [5,6] and have demonstrated potential as solvent polarity indicators for application in bulk optode membranes [7,8,9]. 2,5-Bis((dimethylamino)methylene)cyclopentanone (**4**) and derivatives thereof have been recognized as precursors for the synthesis of symmetrical ketocyanine dyes [8,9,10,11,12].

**Scheme 1:**
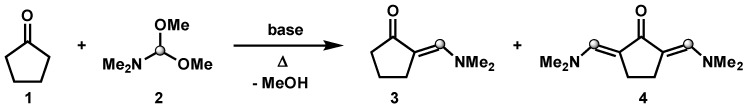
Base-catalyzed condensation of cyclopentanone (**1**) and *N*,*N*-dimethylformamide dimethyl acetal (**2**).

Despite the potentially straightforward approach for the synthesis of this disubstituted cyclopentanone via the condensation of cyclopentanone (**1**) and *N*,*N*-dimethylformamide (DMF)-activated electrophiles (Scheme 1), a detailed and straightforward protocol is yet to be reported. To the best of our knowledge, there are only four reports on the synthesis of compound **4** [10,11,13,14]. In 1983, Tolmachev and co-workers reported the synthesis of **4** through the condensation of **1** and *N*,*N*-Dimethylformamide dimethyl acetal (**2**) [15], using a catalytic amount of 1,5-diazabicyclo(4.3.0)non-5-ene (DBN) in DMF (3.3 M) at 160–195 °C for 10 h to afford the product in 50% yield [10]. Later, in 2004, Takizawa, Akiba and Tani filled a patent where the reaction of **1** and **2** (6.5 equiv) was performed under DBN catalysis (5 mol%) at reflux temperature for five days to give product **4** in 42% yield [13]. In 2008, Callant and Louwet filled a patent where similar reaction conditions were reported to afford product **4** in 39% yield [11]. Therein, DMF was replaced by toluene and 1,8-diazabicyclo [5.4.0]undec-7-ene (DBU) was used instead of DBN. The protocol describes a two-step distillation of methanol: first at 100 °C for 20 h followed by 6 h at 160 °C. In another approach, Zhang and Henry described a less atom-efficient and solvent-free synthesis of similar keto dienamines using bis-dimethylamino-*t*-butoxymethane (up to 4 equiv) as the electrophile at 110 °C [16]. Herein, we describe an operationally simple and solvent-free approach for the synthesis of 2,5-bis((dimethylamino)methylene)cyclopentanone (**4**).

## 2. Experimental Design

### 2.1. Materials

- Cyclopentanone (Alfa Aesar)
- *N*,*N*-Dimethylformamide dimethyl acetal (Fluorochem)
- 1,8-Diazabicyclo[5.4.0]undec-7-ene, DBU (Fluorochem)
- Methyl *tert*-butyl ether, MTBE

### 2.2. Equipment

- Stirring plate (IKA)
- Water circulating system (Julabo)
- NMR (Bruker MX300 spectrometer)

## 3. Procedure

### 3.1. Synthesis of 2,5-Bis((dimethylamino)methylene)cyclopentanone (Time for Completion: 16 h)

1. Add cyclopentanone (0.3 mL, 3.4 mmol), *N*,*N*-dimethylformamide dimethyl acetal (2 mL, 13.6 mmol, 4 equiv) and DBU (50 µL, 0.36 mmol, 10 mol%) to a 10 mL round-bottom flask equipped with a stir bar.
2. Connect a condenser with a circulating water system at 60 °C to the flask and allow the reaction mixture to stir at 190 °C.
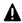
**CRITICAL STEP** The circulating water temperature of the condenser allows the methanol to evaporate from the reaction mixture while condensing the other components (see Appendix A).
  **OPTIONAL STEP** Follow the reaction by ^1^H NMR analysis of crude reaction mixture.
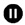
**PAUSE STEP** The heating can be stopped overnight for safety reasons and restarted the next day.
3. After completion (usually 16 h), allow the reaction mixture to cool down to room temperature and observe the crystallization of product (see Appendix B).

### 3.2. Isolation of 2,5-Bis((dimethylamino)methylene)cyclopentanone (Time for Completion: 30 min)

1. Wash the crystals of product with MTBE (3 × 15 mL).
2. Dry the product under vacuum.
  **OPTIONAL STEP** Recrystallize from hot acetone.

## 4. Results

### 4.1. Reaction Optimization

**Scheme 2:**
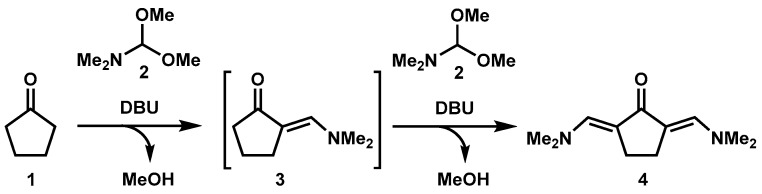
Synthesis of 2,5-bis((dimethylamino)methylene)cyclopentanone (**4**) through base-catalyzed reaction of cyclopentanone (**1**) and *N*,*N*-dimethylformamide dimethyl acetal (**2**). The monosubstituted product—2-(dimethylamino)methylene)cyclopentanone (**3**) is also observed.

The protocol reported herein is a result of a systematic optimization. A crucial step in the aforementioned procedure is the removal of methanol produced during the reaction to shift the equilibrium towards the formation of product **4** (Scheme 2). The study of the selectivity for the formation of mono- and bis-condensation products (**3** and **4**, respectively) was performed by following the reaction by ^1^H NMR spectroscopy.

We started this study by performing the reaction in a closed-vessel reactor for 6 h at 190 °C. Under several different reaction conditions (different solvents, catalytic and stoichiometric amounts of base, etc.), product **3** was exclusively formed (product **4** was never observed by ^1^H NMR analysis of the crude reaction mixture). This result highlights the fact that removing methanol from the reaction mixture is crucial for the success of the protocol. In addition, the use of 4 Å molecular sieves in a closed-vessel reactor also failed to produce **4**, probably because the high temperature displaced the adsorbed methanol back into the reaction mixture.

As the boiling points of methanol, *N*,*N*-dimethylformamide dimethyl acetal (**2**), cyclopentanone (**1**) and 1,8-diazabicyclo[5.4.0]undec-7-ene (DBU) are 65 °C, 103 °C, 131 °C and 261 °C, respectively, we envisioned the use of a condenser with a circulating water system at a temperature close to the boiling point of methanol to allow it to evaporate while keeping the other components inside an open vessel reactor at 190 °C. Gladly, using such a procedure (Section 3) resulted in the formation of product **4**.

Next, we studied the effect of the amount of base and **2** in the reaction. Thus, using a stoichiometric amount of base (2 equiv) resulted in a faster reaction (6 h) compared to that using a catalytic amount of DBU (16 h) as observed by ^1^H NMR analysis of the crude reaction mixture. Despite this faster reaction, the catalytic process is preferential regarding sustainability as no product degradation was observed for extended reaction times. Additionally, the addition of **2** to the reaction mixture divided in two portions (2 equiv added at the beginning followed by 2 equiv added after 6 h) did not seem to improve the reaction progress. Finally, it was observed that the microwave-assisted synthesis did not help in accelerating the reaction progress.

**Figure 1:**
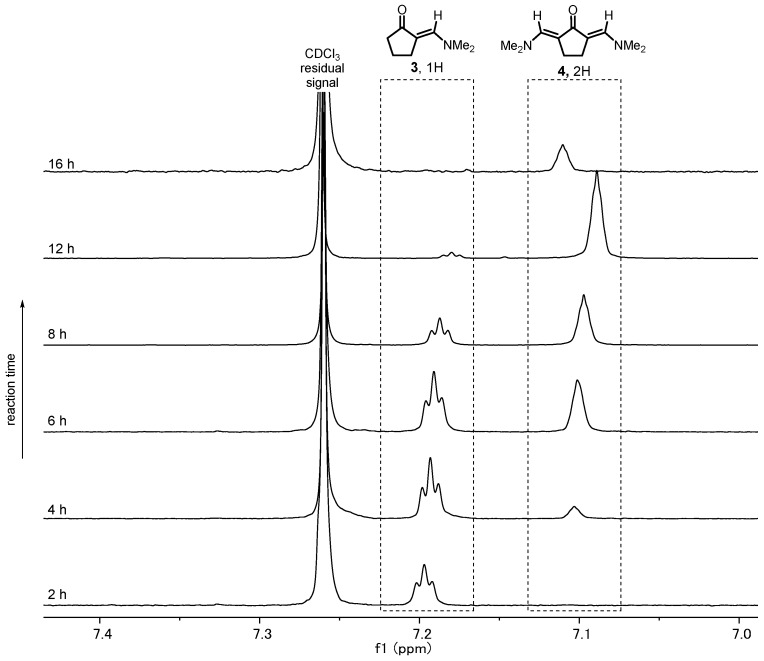
^1^H NMR profile (products **3** and **4**) for the reaction of cyclopentanone (3.4 mmol), *N*,*N*-dimethylformamide dimethyl acetal (13.6 mmol) and DBU (0.36 mmol) at 165 °C for 2 h followed by 14 h at 190 °C.

By performing the protocol reported herein, 100% selectivity of product **4** was obtained after 16 h, as shown in Figure 1. From this crude mixture, the desired product **4** was isolated in 90% yield as a dark crystalline solid. This product can be used as it is for further modifications. The optional recrystallization step of 100 mg of dark crystalline solid resulted in the isolation of 95 mg of a dark yellow solid.

### 4.2. Product Characterization

**Figure 2:**
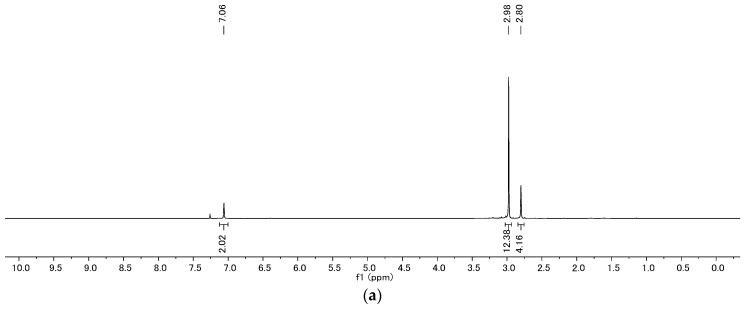

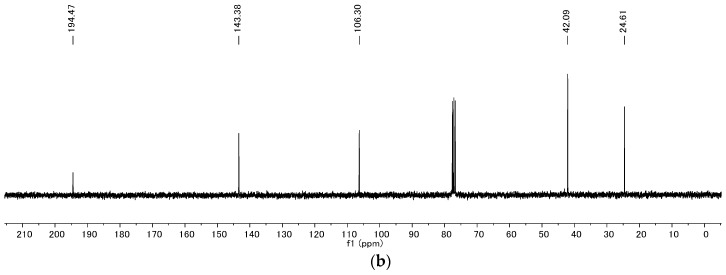
(**a**) ^1^H NMR and (**b**) ^13^C NMR spectra of 2,5-bis((dimethylamino)methylene)cyclopentanone (**4**).

2,5-Bis((dimethylamino)methylene)cyclopentanone (**4**). ^1^H NMR (300 MHz, CDCl_3_) δ 7.06 (s, 2H), 2.98 (s, 12H), 2.80 (s, 4H); ^13^C NMR (100 MHz, CDCl_3_) δ 194.5, 143.4, 106.3, 42.1, 24.6; m.p. 143–144 °C; MS (ESI) *m*/*z* calculated for C_11_H_18_N_2_O [M + H]^+^ 195.15, found 195.05 (Figure 2).

**Figure 3:**
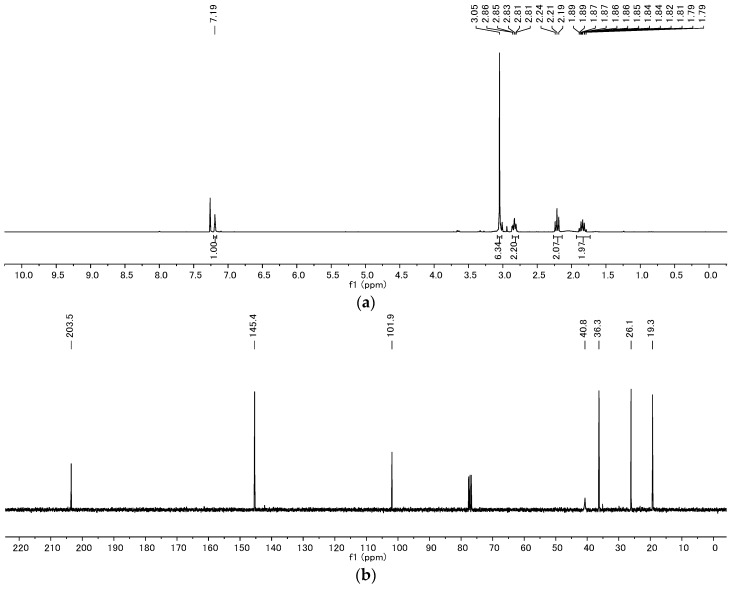
(**a**) ^1^H NMR and (**b**) ^13^C NMR spectra of 2-((dimethylamino)methylene)cyclopentanone (**3**).

2-(dimethylamino)methylene)cyclopentanone (**3**) ^1^H NMR (300 MHz, CDCl_3_) δ 7.19 (t, *J* = 1.7 Hz, 1H), 3.05 (s, 6H), 2.83 (t, *J* = 7.1 Hz, 2H), 2.21 (t, *J* = 7.9 Hz, 2H), 1.83 (apparent quintet, *J* = 7.9 Hz, 2H). ^13^C NMR (100 MHz, CDCl_3_) δ 203.5, 145.4, 101.9, 40.8, 36.3, 26.1, 19.3 (Figure 3).

## Figures and Tables

**Figure A1:**
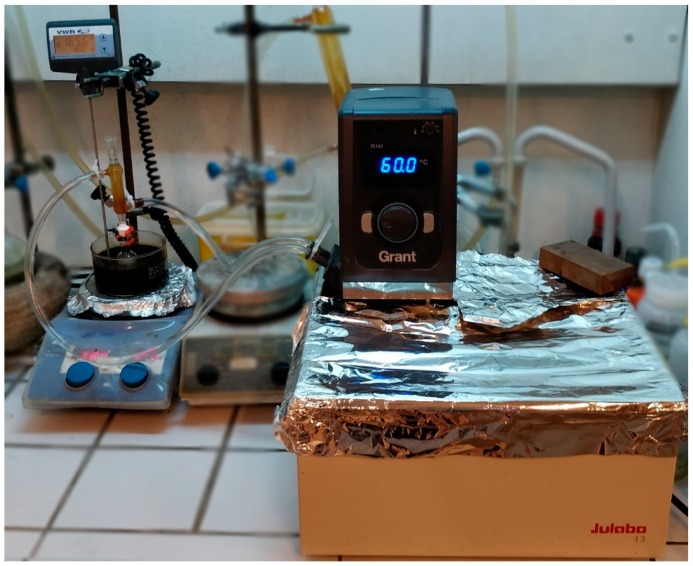
Experimental apparatus for circulating water in the condenser.

**Figure A2:**
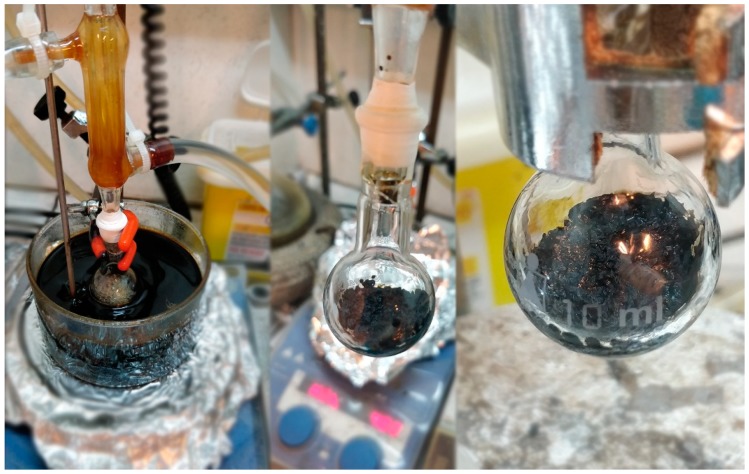
Appearance of the reaction mixture during reflux (**left**) and after cooling down to room temperature (**center** and **right**).
